# Quantification and Gene Expression Analysis of Histone Deacetylases in Common Bean during Rust Fungal Inoculation

**DOI:** 10.1155/2015/153243

**Published:** 2015-12-28

**Authors:** Kalpalatha Melmaiee, Venu (Kal) Kalavacharla, Adrianne Brown, Antonette Todd, Yaqoob Thurston, Sathya Elavarthi

**Affiliations:** ^1^College of Agriculture and Related Sciences, Delaware State University, Dover, DE 19901, USA; ^2^Center for Integrated Biological & Environmental Research (CIBER), Delaware State University, Dover, DE 19901, USA; ^3^Department of Plant Science, South Dakota State University, Brookings, SD 57007, USA

## Abstract

Histone deacetylases (HDACs) play an important role in plant growth, development, and defense processes and are one of the primary causes of epigenetic modifications in a genome. There was only one study reported on epigenetic modifications of the important legume crop, common bean, and its interaction with the fungal rust pathogen* Uromyces appendiculatus* prior to this project. We measured the total active HDACs levels in leaf tissues and observed expression patterns for the selected HDAC genes at 0, 12, and 84 hours after inoculation in mock inoculated and inoculated plants. Colorimetric analysis showed that the total amount of HDACs present in the leaf tissue decreased at 12 hours in inoculated plants compared to mock inoculated control plants. Gene expression analyses indicated that the expression pattern of gene* PvSRT1* is similar to the trend of total active HDACs in this time course experiment. Gene* PvHDA6* showed increased expression in the inoculated plants during the time points measured. This is one of the first attempts to study expression levels of HDACs in economically important legumes in the context of plant pathogen interactions. Findings from our study will be helpful to understand trends of total active HDACs and expression patterns of these genes under study during biotic stress.

## 1. Introduction

Histone deacetylases are a family of enzymes that remove acetyl groups from lysine residues present in the N-terminal extension of core histones of nucleosomes [1] and have been found in bacteria, fungi, plants, and animals. Histone acetyltransferases (HATs) and deacetylases (HDACs) play an important role in chromatin structural modifications and epigenetic changes in many organisms. Research on histone deacetylase inhibitors (HDAC) began nearly 30 years ago when studies were laid out to understand why dimethyl sulfoxide (DMSO) caused terminal differentiation of murine erythroleukemia cells [2]. This early observation led to the development of novel pharmacological agents in the field of chromatin remodeling [1]. HDACs catalyze deacetylation reactions, which cause chromatin to coil by removing acetyl groups from lysine residues of histones. This deacetylation increases the positive charge on N-termini of the core histones. As a result, the interaction between core histones and negatively charged DNA increases which causes tight coiling of DNA, which in turn blocks access to the transcriptional machinery. The balance between the actions of HDACs, HATs, and transcriptional elements serves as a key regulatory mechanism for gene expression and in turn governs numerous developmental processes and disease states [3, 4].

HDACs are known to be involved in a myriad of plant physiological and developmental activities and in epigenetic events often for transcriptional repression of genes [5, 6]. Several studies in plants have reported that there is a direct correlation of DNA methylation, histone deacetylation, and gene suppression [5, 7, 8]. In the model plant,* Arabidopsis thaliana*, HDA6 and MET1 interact directly to silence transposable elements by modifying DNA methylation, histone acetylation, and histone methylation status [9, 10]. Genetic analysis in* Arabidopsis* indicates that HDA6 is a component of RdDM (RNA-directed DNA methylation) pathway [7]. Even in other systems like the African clawed frog* Xenopus laevis*, relaxation of methylated DNA in oocytes by the inhibition of histone deacetylases was observed [8, 11].

Removal of acetyl groups from histones at promoter regions chiefly correlates with gene silencing and transcriptional repression. However, previous studies have also shown gene repression [5, 12] as well as activation of some genes [13]. Hence, the specificity of HDACs for regulation of distinct gene programs depends on cell identity (cell state identified by gene regulation programs) and the scale of available partner proteins along with the signaling networks of the cell [13, 14]. As an example, HDA6 in* Arabidopsis*, by interacting with different proteins, can regulate flowering time [15, 16], leaf development [17], transposon silencing [18], salt and ABA stress [19], ethylene and jasmonate signaling [20], and freezing tolerance [21]. Gene HDA19 can regulate seed maturity [22], flowering time [23], immune response, and seed dormancy by interacting with other proteins [24, 25]. HDA9 has also been reported to regulate flowering in* Arabidopsis *by repressing the floral activator AGL19 [26]. Additionally, HDA6 was shown to be involved in histone modifications by increasing gene expression in* Arabidopsis* during seed germination, salt stress, and abscisic acid treatments [27]. HDACs also showed response to various biotic stresses. In* Arabidopsis*, HDA19 showed induced expression when plants were challenged with* P. syringae* and the stability of induced transcripts was shown to be dependent on the levels of salicylic acid and pathogen-related 1 (NPR-1) gene expression [28].

Recent phylogenetic analysis of sequences from the HDACs superfamily RPD3/HDA1 from* Arabidopsis* enabled further classification into three classes, class I, class II, and class III [29]. Similarly, genome analysis of rice HDACs enabled the identification of an additional class, class IV, [30] indicating the diversity and need for further studies in other commercially important crop plants including legumes. Expression analysis of HDACs from all classes and families showed differential expression during developmental stages, environmental stresses, and hormonal stimuli [31, 32].

The long-term goal of our research is to understand epigenetic modifications in common bean during infection by the rust fungal pathogen. In this study, we report progress on our understanding of the role of HDACs during infection of common bean with the bean rust pathogen,* U. appendiculatus* race 53. We focus on understanding and quantifying total HDAC activity present in mock inoculated (MI) and inoculated (I) leaf tissues at 0, 12, and 84 hours after inoculation (hai) and analyses of the expression profiles of selected genes from each known plant HDAC family.

## 2. Materials and Methods

### 2.1. Plant Materials and Pathogen Infection

**Figure 1:**
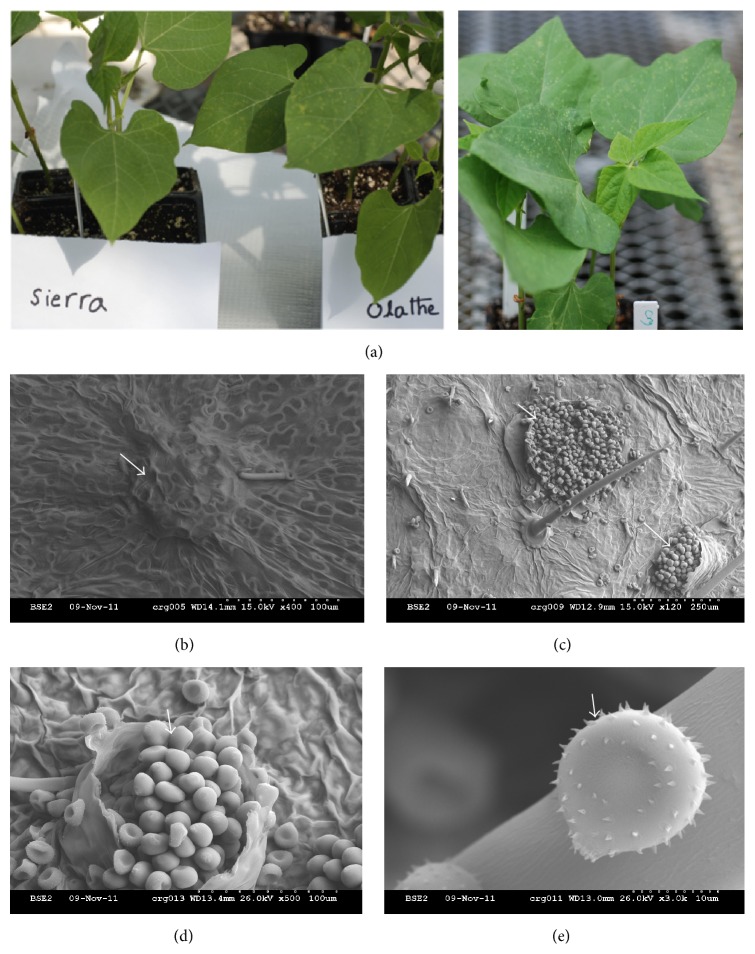
Common bean rust symptoms, rust pustules, and spores. (a) Ten-day-old seedlings at the primary leaf stage were inoculated with rust race 53. The susceptible genotype Olathe and susceptible mutant crg developed visible rust like symptoms after approximately 10 days of inoculation, whereas the resistant genotype Sierra was asymptomatic as expected. The genotypes Olathe and crg were used as an inoculation experimental control only and are shown here. (b–e) Scanning electron microscope pictures from a leaf of a rust susceptible genotype crg after symptoms were developed. Picture (b) is an unopened rust pustule. ((c) and (d)) Pustules which burst open at 120 and 500 times' magnification. (e) Rust spore under 3000 times' magnification. White arrows point to the pustules and fungal spores.

The common bean cultivar “Sierra” is resistant to common bean rust race 53 (Figure 1(a)) and carries the rust resistant genes* Ur-3* and* Crg* [33, 34]. Sierra exhibits a hypersensitive response upon inoculation with race 53, the cultivar “Olathe,” which is recessive at* Ur-3* is susceptible to this pathogen race. The genotype crg, a susceptible mutant derived from Sierra which carries a mutation at the* Crg* locus also develops rust like symptoms (rusty-yellow or bright orange spots) on leaves. Both Olathe and crg were used in this experiment as control for demonstrating successful inoculations.

**Figure 2:**
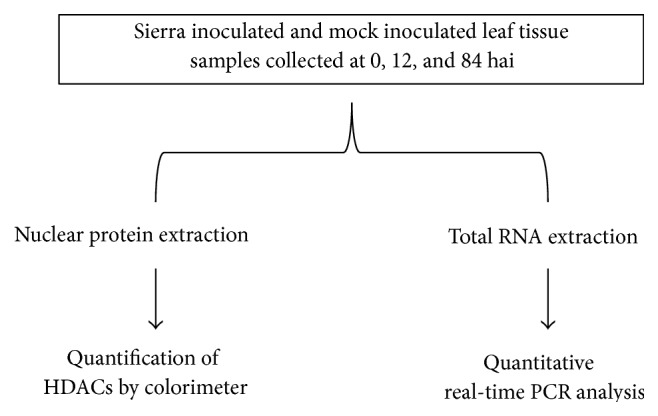
Flowchart outlining the experiment in this study. Same sets of materials from each biological replicate were utilized to perform colorimetric analysis and gene expression studies.

Plants were grown in the greenhouse as per Melmaiee et al. [35]. When plants were ten days old at the primary leaf stage, half of the seedlings from each genotype were inoculated with* U. appendiculatus* race 53 spores with 1% Tween 20 on the adaxial and abaxial sides of the two leaves and another half of the seedlings were mock inoculated (MI) with only 1% Tween 20 along with Olathe and crg as inoculation experimental controls. After inoculation, plants were placed in a growth chamber with high humidity (approximately 90%) to facilitate the establishment of fungal growth. Sierra leaf samples were collected at 0, 12, and 84 hai along with MI samples for analysis as shown in Figure 2 for nuclear extraction and total RNA isolation. The above time points were chosen based on our previous experiments [35, 36]. For each sampling time, leaves were pooled from three different plants (one leaf from each plant) and utilized for colorimetric assays, and three leaves from another set of three plants were collected and flash frozen for gene expression analyses. The entire experiment was repeated twice yielding two biological replications of the study.

### 2.2. Scanning Electron Microscopy

Symptomatic leaves were collected from susceptible mutant genotype crg (derived from Sierra) [33], dehydrated with ethanol, and mounted on stubs using carbon filled adhesive. The dehydrated specimens were coated with gold palladium by sputter coater 108 auto (Cressington Scientific Instruments Ltd., Watford, UK) and observed with an analytical scanning electron microscope S-2600N (Hitachi High Technologies America, Inc., Schaumburg, IL) located in the College of Agriculture & Related Sciences at DSU.

### 2.3. Nuclear Extraction

Nuclear extractions were carried out from MI and I leaf samples using the EpiQuik Nuclear Extraction kit 1 (Epigentek Group Inc., Farmingdale, NY). Approximately one gram of leaf samples (either flash frozen or fresh) was cut into small pieces and submerged in a 1 : 10 diluted nuclear extraction buffer 1 (NE1) with 1x dithiothreitol (DTT) in a mortar and ground thoroughly until all the leaf samples became fine paste. Samples were incubated on ice for 15 minutes and centrifuged for 10 minutes at 11,000 ×g to obtain a nuclear pellet. The supernatant was removed and 500 *μ*L of nuclear extraction buffer 2 (NE2) containing 1x DTT was added to the nuclear pellet and incubated for another 15 minutes on ice. During this time samples were vortexed for 5 sec at three-minute intervals to increase nuclear protein concentration. Samples were then centrifuged at 14,000 g for 10 minutes at 4°C, and the nuclear protein was quantified with Qubit fluorometer (Life Technologies, Grand Island, NY) and stored at −80°C for further analyses.

### 2.4. Quantification of HDACs by Colorimetric Method

HDAC activity was measured with 1.545 *μ*g of nuclear protein extract following the manufacturers protocol (HDAC Assay Kit, colorimetric, Active Motif, Carlsbad, CA). As suggested in the protocol for samples with potentially low HDACs, we extended the initial HDAC reaction time to three hours. Since the kit was developed based on nuclear extracts from mammalian cells, we envisioned that extending the incubation time will help complete the deacetylation reaction. Samples were measured in triplicate (standards were measured in duplicate) using EPOCH colorimetric plate reader (Biotek, Winooski, VT) at 405 nm. In the assay reaction, a short peptide substrate was added along with the nuclear extract and other reagents as per the protocol. This substrate contains acetylated lysine residues and can be deacetylated by most HDAC enzymes. Active HDACs from the experimental samples would then bind to the added substrate by removing acetyl groups from the substrate. This reaction then yielded an HDAC-deacetylated colored product, which was measured by the colorimetric plate reader. The amount of deacetylated product in the reaction is directly proportional to the amount of active HDAC enzymes present in our samples [37].

### 2.5. Selection of HDAC Gene Sequences and Primer Design

Representative proteins from each HDAC family or class were selected based on the previous reports [29]. The GenBank protein accession numbers AAC50038, AAK0712.1, BAB10553, NP_200914, NP_200915, and AAD40129, from RPD3 (reduced potassium dependency)/HDA1 (histone deacetylase 1) family, AAB70032 from HD2 family, and BAB09243 from SIR2 (Silent Information Regulator 2) were selected for gene expression analysis. All of these sequences were derived from* Arabidopsis thaliana* except AAK01712.1 and AAC50038, which were derived from rice (*Oryza sativa*) and maize (*Zea mays*).

GenBank protein accession numbers were used to extract model organism protein sequences from NCBI database and these sequences were compared against the common bean predicted proteome derived from the common bean genome-sequencing project from http://www.phytozome.org/ [38]. The best match was selected and coding sequences (CDS) were extracted for further analysis (Supplementary File 1) (see Supplementary Material available online at http://dx.doi.org/10.1155/2015/153243). Proteins AAK0712.1 and AAC 50038.1 matched the same common bean CDS Phvul.009G115300.1 and proteins NP_200914 and NP_200915 matched Phvul.003G185200.1 and other proteins matched different common bean sequences as shown in Table 1. For convenience, we named these CDS (referenced in this study as* Phaseolus vulgaris* HDACs) as mentioned in column 4 of Table 1. For gene expression analysis, primers were designed with Primer quest software as in Table 2 and tested with common bean genomic DNA (Figure 4(a)).

### 2.6. Total RNA Isolation and cDNA Synthesis

Total RNA was extracted using TRIzol reagent (Invitrogen, Carlsbad, CA) from flash frozen pooled leaf tissues (three leaves from three plants) as per manufacturer's protocol and the RNA was digested with the enzyme rDNAse (Life Technologies, Grand Island, NY) to remove any contaminating DNA. Absence of genomic DNA was confirmed with known primers that can amplify intronic regions as mentioned previously [39]. Total RNA was used to synthesize cDNA with ProtoScript M-MuLV First Strand cDNA synthesis kit (New England BioLabs, Beverly, MA).

### 2.7. Gene Expression Analysis by Quantitative Real-Time PCR (qRT-PCR)

Concentrations of cDNA were equalized for all the samples under consideration and qRT-PCR analysis was carried out on Applied Biosystems 7500 real-time machine (Foster City, CA) using SYBR Green dye. Gene expression was normalized to the housekeeping gene ubiquitin-conjugating enzyme E2 UBC9 (TC362) [40] and included in each PCR run. The whole experiment was replicated twice with three technical replications for each sample analyzed. Gene expression analysis was carried out by comparative 2^−ΔΔCT^ method [41] and used to calculate expression values and indicated in fold changes. Student's *t*-test was performed with a *P* value cutoff of 0.05.

## 3. Results and Discussion

### 3.1. HDACs Activity during Fungal Infection

**Figure 3:**
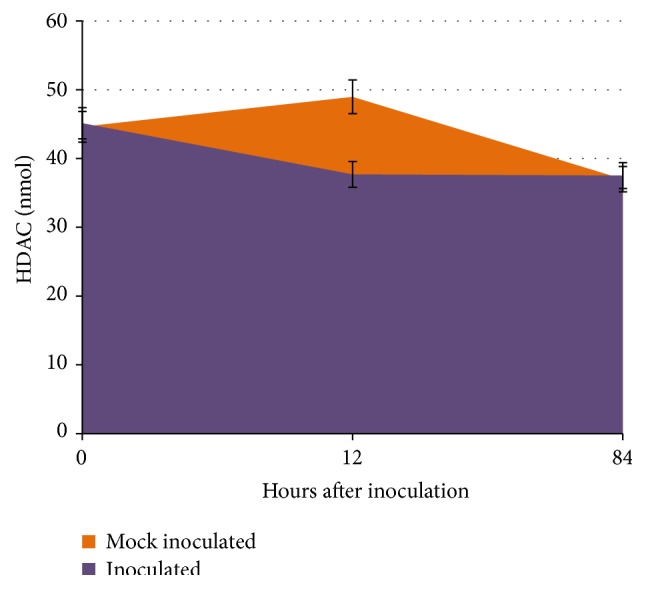
HDAC activity between mock inoculated and inoculated common bean. Total HDAC activity was measured based on the optical density (OD) and the amount of activity was determined based on the standard curve. The experimental values differ significantly with a probability value of 0.05%. The error bars represent standard deviation.

Activity of total HDAC enzymes was quantified by a colorimetry-based assay. We collected leaf tissue from inoculated and mock inoculated rust resistant genotype Sierra (tissue pooled from 3 leaves for each time point) at 0, 12, and 84 hai, from which nuclear extracts were then isolated and processed. A standard curve was generated using the standards provided with the HDAC Activity Kit and optical density (OD) values of the samples from MI plants and I plants were then extrapolated. The mean values from two independent biological replicates were calculated (Figure 3). Colorimetric analysis revealed that there is a reduction in the amount of active HDACs (37.68 nmol) in the inoculated samples at 12 hai compared to mock inoculated plants (48.97 nmol), whereas at 84 hai the activity was approximately 37.0 nmol in both the samples.

The reduction in overall HDAC activity at 12 hai suggests that there may be less deacetylation reactions at this time point and more uncoiled DNA was available for transcription as there will be a demand for induction of stress resistant genes at this time. However, colorimetric analysis indicates that HDACs activity changes throughout the course of rust infection in common bean plants and differs between plants challenged or not challenged with the fungal pathogen.

### 3.2. Identification of Common Bean Homologous Sequences

**Figure 4:**
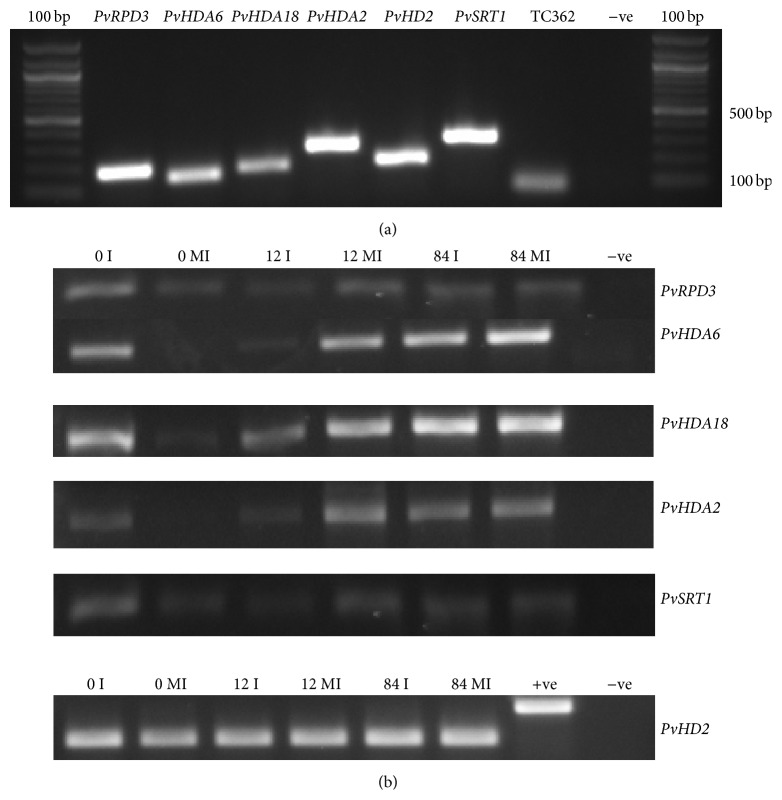
PCR amplification of HDAC genes under study. (a) Primers for the selected genes were amplified by PCR with Sierra genomic DNA and electrophoresed on a 2% agarose gel. In positive (+ve) control, reference gene TC362 primers were used and in negative (−ve) control no primers were added to the PCR reaction. (b) The same HDACs primers were tested with experimental cDNA by PCR amplification and electrophoresed on a 2% agarose gel. In +ve control, genomic DNA was used instead of cDNA and in −ve control no primers were added to the PCR reaction.

HDAC protein sequences were obtained from* Arabidopsis* and other plant species from GenBank using corresponding protein accession numbers. These protein sequences were searched against the common bean predicted protein database and common bean CDS were obtained (Supplementary File 1) for gene expression analysis. Since common bean CDS were derived by bioinformatics analysis, corresponding primers were initially amplified with genomic DNA of the common bean (Figure 4(a)) and then with cDNA derived from the experimental samples (Figure 4(b)), which were used for qRT-PCR analysis. All the genes tested were amplified in both the genomic DNA and cDNA samples.

### 3.3. Gene Expression Analysis

**Figure 5:**
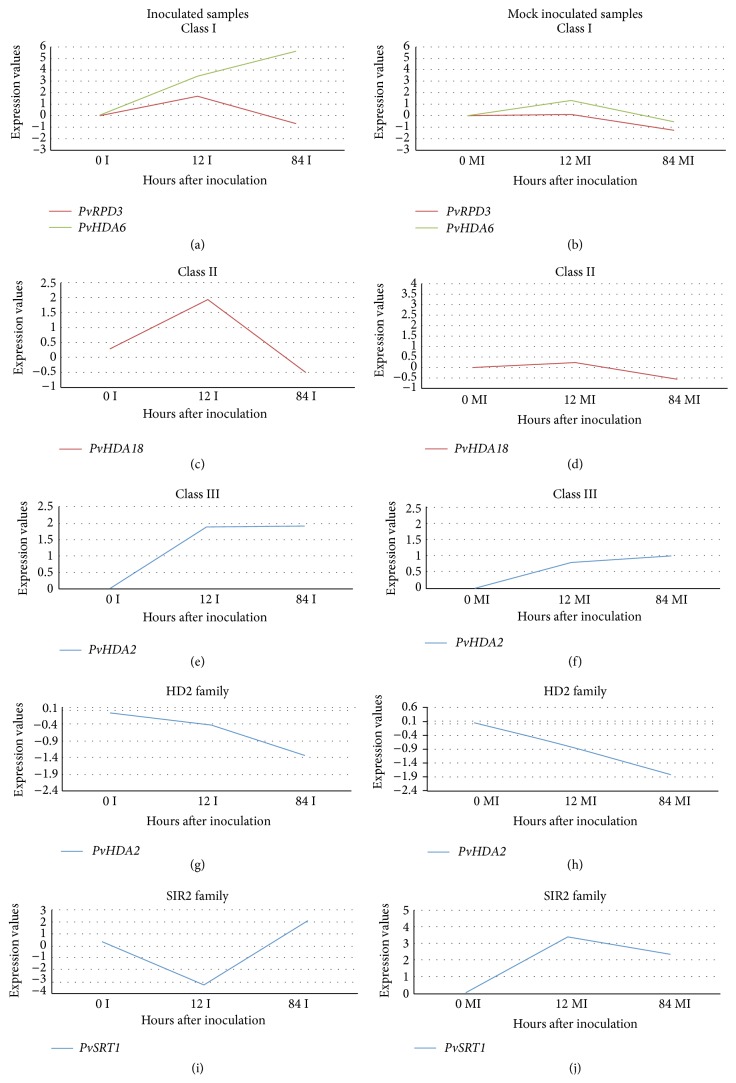
qRT-PCR analysis of HDAC genes. Figures on the left-hand side are from inoculated samples, while figures on the right-hand side are from mock inoculated samples. Sampling time points are shown in *x*-axis and ΔΔCT values are shown in *y*-axis. Sierra 0 hai mock inoculated samples and the endogenous gene TC362 were used for calculating expression values.

Between the two genes that were studied in class 1 (RPD3 family), gene* PvHDA6 *showed increased expression at both 12 and 84 hai in the inoculated samples (Figure 5(a)). In the MI samples, the* PvHDA6* expression was seen to be slightly increased at 12 hai and was neutral at 84 hai samples (Figure 5(b)).* PvRPD3* expression was seen to be slightly increased at 12 hai in the I samples. However, both the genes showed slight reduction in expression at 84 hai in the MI samples. Gene* PvHDA18* from class II HDACs showed increased expression at 12 hai and reduced expression at 84 hai in MI and I samples (Figures 5(c) and 5(d)). Class III gene* PvHDA2* showed increased expression at both 12 and 84 hai in I samples whereas its expression was neutral in MI samples at both the time points (Figures 5(e) and 5(f)).

Similar results as observed in this study were seen in a* Pseudomonas syringae* resistant* Arabidopsis* plant with RPD3/HDA class gene* HDA19*. Increased expression levels of* HDA19* were seen when plants were inoculated with the bacterial pathogen pstDC3000, a virulent strain of* P. syringae pv. tomato* [28].* HDA19* by interacting with the transcription factors* WRKY38* and* WRKY62* was suggested to help fine-tune basal defense responses to pathogen attack in* Arabidopsis* [28]. Contrastingly, Choi et al. [42] showed that* HDA19* played a negative role in basal defense response mediated by salicylic acid-dependent signaling pathway, where they have observed increased expression of pathogen defense genes in* HDA19* mutant plants.

In our analysis, gene* PvHD2* was neutral in its expression at 12 hai and showed decreased expression at 84 hai in both MI and I leaf samples (Figures 5(g) and 5(h)). In a recent report, the tobacco* NtHD2a* and* NtHD2b* genes showed a rapid and strong reduction in their expression after treating the tobacco cells with cryptogein, an elicitor of tobacco defense and cell death [43].

Based on earlier findings, the HD2 class is plant specific and found only in plants [29, 44]. Differential expression of the barley HD2 genes (*HvHDAC2* and* HvHDAC2-2*) was observed in different tissues and during seed development in barley [32] and they also exhibited differential expression in barley cultivars with varying seed size. In the same study, these genes responded to plant stress hormones such as jasmonic acid (JA), abscisic acid (ABA), and salicylic acid (SA) suggesting a possible role in epigenetic regulation due to biotic and abiotic stresses and during seed development.

Gene* PvSRT1* from the SIR2 family showed contrasting expression at 12 hai, for which the expression levels were decreased in I samples and increased in MI samples. However we observed that the expression levels were increased at 84 hai in both the samples as in Figures 5(i) and 5(j).* SIRT1* was reported to regulate miRNA in Alzheimer's disease patients [45, 46]. SIR2 genes were found to be highly expressed in highly proliferating stages such as the seedling and developing panicle stages [47]. In the current study, we have used 10-day-old common bean seedlings for inoculation; hence this might be a possible reason for the presence of a higher quantity of SIRT proteins overall. This may be why the trends of active HDACs and the expression profiles of* SIRT* gene are similar. Additionally, we note that in this study we were able to measure one representative gene (*PvSRT1*) from this class, and it will be interesting to measure the second gene. Pandey et al. [29] pointed out that only two genes from the SIRT class are currently known in plants in this class. Another consideration for future quantification experiments would be to determine protein turnover changes.

Reduced expression of the rice SIR2 family gene* OsSRT1* by specific RNA interference increased histone H3K9 acetylation, decreased H3K9 dimethylation, and also led to the development of cell death and symptoms related to plant hypersensitive response during incompatible interaction with pathogen [47]. Interestingly, in our study,* PvSIRT1* showed decreased gene expression at 12 hai in leaves of inoculated plants in the bean genotype that also exhibits hypersensitive response.

HDACs play an important role in plant growth, development [48], flowering, seed maturity, and defense/tolerance to biotic and abiotic stresses. Each HDAC gene has unique functions and these genes substitute or complement each other's function. A recent observation indicated that rice HDAC genes showed more divergent functions than their homologs in* Arabidopsis* [49] and the same study also showed that their expression is tissue/organ specific in rice.

Based on the available literature, HDAC genes interact with histone and nonhistone proteins as well as other regulatory elements. HDA6 has been reported to interact with small interfering RNAs (siRNAs) that are generated through the RdDM pathway to suppress gene activity [50, 51]. HDA9 has been reported to regulate flowering in* Arabidopsis* by repressing flower-activating gene AGL 19 [26]. Histone deacetylase HDA6 is required for freezing tolerance [7]. HDA19 by interacting with WRKY 38 and WRKY62 showed enhanced basal resistance to bacterial pathogen [28].

In conclusion, reduced total HDACs activity was observed at 12 hai in rust inoculated bean plants compared to mock inoculated plants. Majority of the RPD3/HDA1 family of HDACs studied showed increased expression at least in one time point observed after inoculation. The* PvHD2* gene of plant specific HDACs did not show differential expression with inoculation and may possibly be developmentally regulated. Additionally, the* PvSIRT1* gene showed reduced expression at 12 hai in inoculated samples. Epigenetic analysis in common bean itself is in its infancy. This is one of the first attempts to try to understand HDACs gene regulation in common bean. As HDACs play important roles in chromatin modification, in normal plant developmental process, and in biotic/abiotic responses, our findings can be helpful to study other commercially important legume crops.

## Supplementary Material

The supplementary file contains model organism HDAC protein IDs studied in this project and predicted corresponding coding sequences obtained from common bean genome (http://phytozome.jgi.doe.gov/pz/portal.html). Location of these coding sequences on common bean genome was mentioned based on the bioinformatics analysis.

## Figures and Tables

**Table 1:** Selected representative HDAC genes for expression analysis. GenBank protein accession number and corresponding predicted common bean homologs along with location on the common bean genome.

Gene family/class	GenBank protein accession number	Phytozome common bean CDS number	HDACs names	Location on the bean genome (chromosome)	Corresponding model organism
RPD3/HDA1 family class I	AAC50038 AAK0712.1	Phvul.009G115300.1	*PvRPD3*	9	*Zea mays* *Oryza sativa*
	BAB10553	Phvul.003G203800.1	*PvHDA6*	*6*	*A. thaliana*
Class II	NP_200915 NP 200914	Phvul.003G185200.1	*PvHDA18*	*3*	*A. thaliana*
Class III	AAD40129	Phvul.001G034500.2	*PvHDA2*	*1*	*A. thaliana*
HD2 family	AAB70032	Phvul.001G186300.1	*PvHD2*	sg0.contig 03923: 2769–5777	*A. thaliana*
SIR2 family	BAB09243	Phvul.006G057700.1	*PvSRT1*	*6*	*A. thaliana*

**Table 2:** Primer sequences utilized for qRT-PCR.

Predicted common bean gene	Primer sequences
Phvul.009G115300.1	ACATGAGCGTGTTCTGTACGTGGA TCAGCACCGCATTGGAGAACTACT
Phvul.003G203800.1	CATCCGCATGGCGCACAATCTTAT ACCCAACCTGTCACCAGACAATGA
Phvul.003G185200.1	TCTGCGGTTAGTGCATTCCAGAGT GGGTCACCAACAGCTGCATCAAAT
Phvul.001G034500.2	TCGGCATAGAGAAACTGCATCCGT ACCACCTGAAGTGAGCATGACGAT
Phvul.001G186300.1	AACTGGTAGCCCTGAACGTGAAGT TCCCATTGGCAGCACTAACTGGAA
Phvul.006G057700.1	CTTGCCAGAAGCATCACTGCCATT GGCAAGTTGCACGCTGGAGTTATT
TC362	GCTCTCCATTTGCTCCCTGTT TGAGCAATTTCAGGCACCAA
