# Supplementary material for: Quantification and Gene Expression Analysis of Histone Deacetylases in Common Bean during Rust Fungal Inoculation
Source: Int J Genomics. 2015 Dec 28;2015:153243. doi: 10.1155/2015/153243 (PMC4707378; doi:10.1155/2015/153243)
Supplement: Supplementary file 1 — The supplementary file contains model organism HDAC protein IDs studied in this project and predicted corresponding coding sequences obtained from common bean genome (http://phytozome.jgi.doe.gov/pz/portal.html). Location of these coding sequences on common bean genome was mentioned based on the bioinformatics analysis. [file 153243.f1.docx]

**1. BAB100553 ATHDA6**

>Phvul.003G203800.1 CDS
ATGGGTATGATGGAAGAGGAGAGTAGCAATAGCAACATAGAAGGTGGGGCTTCGCTGCCATCGTCAGGTTCCGACGCCAAAAAGCGAAGAGTCACGTACT
TTTACGAACCAACTATCGGCGATTACTACTACGGCCAGGGCCACCCAATGAAGCCGCACCGCATCCGCATGGCGCACAATCTTATCGTCCACTACTCCCT
CCACCGCCGCATGGAGATTAACCGCCCTTTCCCGGCCTCCACCGCCGACATTCGCCGCTTTCACTCCGACGACTACGTCCACTTCCTCTCTTCCGTCTCC
CCCGAGACCCTTTCGGACATCACCTTCTCGCGCCAACTCAAACGCTTCAACGTCGGCGAGGACTGCCCTGTCTTCGACGGCCTCTTCGACTTCTGCCAAG
CTTCCGCTGGAGGGTCCATCGGCGCCGCCGTCAAACTCAACCGCGGTGACGCTGACATCGCCATCAATTGGGCCGGCGGCCTCCACCACGCCAAGAAGTC
CGAAGCCTCTGGATTCTGTTACGTCAACGACATTGTTCTTGGTATCCTCGAGCTTCTCAAAGTTCACAGGCGAGTTCTGTATGTTGACATTGATGTTCAC
CATGGTGATGGAGTTGAGGAGGCCTTTTACACCACTGATAGAGTAATGACAGTCTCTTTTCACAAGTTTGGGGACTTTTTCCCTGGCACAGGGCATATCA
AAGACATTGGGGTGGGCTCGGGAAAGAATTATGCGGTCAATGTCCCATTAAACGACGGAATGGATGATGAGAATTTTCGTGGTCTGTTTCGACCTATCAT
TCATAAAGTCATGGAGGTTTATCAACCTGAGGCAGTTGTTCTTCAATGTGGAGCTGATTCATTGTCTGGTGACAGGTTGGGTTGCTTCAACTTGTCTGTG
AAAGGTCATGCAGATTGCCTTCGATTCCTTAGATCTTTCAATGTTCCTTTAATGGTTTTGGGTGGGGGTGGATATACAATTCGGAATGTTGCCCGTTGTT
GGTGTTATGAGACAGCAGTGGCAGTAGGAGTGGAGCCTGACAATAAGTTGCCTTATAATGAATATTATGAATATTTTGGTCCAGATTATACTCTCTATGT
CGATCCAAGCAACATGGAGAACCTAAACACACCCAAGGATATGGAAAAAATAAGGATCACACTACTAGAACAGATATCCCGTCTTCCCCATGCTCCCAGT
GTACCTTTTCAGACAACACCACCTACCTTAGAAATTCCAGAAGAGGCTGAAGAGGACATGGATAGAAGACCAAAACTTCGCAAATGGGATGGTGAAGATT
ATGATTCTGACCCTGATGAAGGTGGAAAGGCTAATTCCAAGTTCTCAAATGTCAATGCCCATATGAGGGAAATTGTAGATGACATGGAAGAAGAGAAGCC
AGGAGTGCATCCACCGTCTTGTTGTTGA

***Location:*** Chromosome 6

**2. zmRPD3 AAC50038.1**

>Phvul.009G115300.1 CDS
ATGGATTCCGGCGGCAATTCTTTACCGTCTGCACCTGATGCGGTTAAGAGAAAGGTTTGCTATTTCTATGATCCAGAGGTTGGGAATTACTATTATGGTC
AAGGTCACCCTATGAAGCCACATCGTATACGGATGACACATGCTCTTCTTGCTCATTATGGATTGCTTCAGCACATGCAGGTCCATAAGCCATTTCCTGC
TAGAGATCGGGATCTTTGTCGTTTTCACGCTGATGATTATGTTGCATTCCTTCGAAGCATAACTCCTGAAACACAGCAGGATCATCTGAGGCAACTCAAA
CGCTTTAATGTTGGTGAAGATTGTCCTGTTTTTGATGGCCTCTACTCTTTCTGTCAAACTTATGCAGGGGGGTCAGTTGGGGGTGCTGTCAAGTTAAATC
ATGACCAATGTGATATTGCTGTCAACTGGGCTGGTGGGTTGCATCATGCCAAGAAGTGTGAGGCTTCTGGATTTTGCTATGTTAATGATATAGTCCTCGC
AATCTTAGAACTTCTTAAACAACATGAGCGTGTTCTGTACGTGGATATTGATATCCACCATGGAGATGGTGTGGAGGAAGCATTCTATACTACTGACAGA
GTCATGACTGTTTCTTTTCATAAATTCGGAGACTATTTTCCTGGTACTGGGGATGTACGTGATATTGGATATGGAAAAGGAAAATACTACTCTCTTAATG
TTCCACTTGATGATGGCATTGATGATGAGAGTTATCATTTCTTGTTCAAACCATTAATTGGCAAAGTAATGGAAGTGTTCAGACCTGGTGCAGTAGTTCT
CCAATGCGGTGCTGATTCTCTATCTGGAGACCGATTGGGATGTTTCAATCTTTCAATTAGGGGACATGCAGAGTGTGTCAAATACATGAGATCATTCAAC
GTGCCCCTCTTGCTACTAGGTGGTGGTGGCTACACCATTCGGAATGTTGCTCGATGTTGGTGCTATGAGACAGGAGTTGCTCTTGGAATAGAAGTTGATG
ACAAAATGCCAGAACATGAGTATTATGAATATTTTGGTCCAGATTATACCCTCCATGTTGCCCCAAGTAACATGGAAAACAAGAATTCCCGTTATTTACT
TGAAGAAATTCAATCTAAGCTACTTGAGAATCTTTCCAAGCTGCAGCATGCTCCTAGTGTCCAATTTCAGGAAAGGCCTCCGGATTCTGATCTTGGAGAG
GCAGATGAAGATCATGACAATGGAGATGAGACATGGGATCCAGATTCAGACATGGACGTTGATGTTGAACGTGGGCTTGTACCAAGCAGAGTAAAGAAAG
AAATAGTTGAACCGCAACTCAATGATCCGGATGACCAAAGAAGAAGTGGGGAGCATTTGAGAGACTCTGATACTGCAGTTGCTGAAACTTGTATGAAGGC
TCTAGATATTTCTTCTCAGAAAGCTGATGAAGATAATGTGAAAGTTGAACACAATACTGTGAATGATTTGACCAAAGAGACGGGCCTAAAGTGCTAA

***Location:*** Chromosome 9

**3. NP_200915**

>Phvul.003G185200.1 CDS
ATGGAGGGCGAGAGTGCGAAGAAGAGTAGCGAAAGCAGTGTGAACGGTCAACCCCGTGTGGGTTTACTATACGATAGGAGAATGTGCAAGCACCACACGC
CGAACAACGAGGACCACGTCGAGACTCCTAATCGCATTAGGTCAACTTGGAACCACCTCGAGAGCGCCGGCATTCCTCAACGATGCCTGATTCTGGAGGC
CAAGAAAGCTGAAGACAAACATCTGCGGTTAGTGCATTCCAGAGTTCATGTAAATCTGATTAAGAACATTAGCTCCAAGCAATTCAGTTCGCGGAGACCC
GAGATTGCCTCCAAATTGAATTCCATATACTTTAACGAAGGTTCATCTGAAGCTGCGTACCTTGCTGCTGGCTCTGCTGTAGTGGTTGTTGAAAAAGTGG
CAAGCGGGGAATTGGATTCGGCTGTTGCCATTGTTAGGCCTCCAGGTCATCATGCAGAACAGAATGAAGCTATGGGGTTTTGTCTCTTCAACAATGTGGC
AGTTGCTGCAAGATATCTCTTAGACGAAAGACCTGATTTAGGTGTGAAGAAAATTTTAATTGTTGATTGGGATGTCCATCATGGAAATGGTACTCAAAAG
ATGTTCTGGAATGACTCTCGAGTTTTATTCTTCTCCGTTCACAGGCATGAGTTTGGGAGTTTTTATCCTGCTAATGACGATGGATTTTATACTAAGGTGG
GAGAAGGAGAAGGTGCTGGATATAATATAAATGTCCCCTGGGAGAATGGGAGATGTGGTGATGCAGATTACTTTGCAGTGTGGGACCACATTTTGCTTCC
TGTAACCAAAGAATTTAATCCAGACATAATTATAGTTTCCGCGGGATTTGATGCAGCTGTTGGTGACCCTTTGGGAGGATGTCGTGTCACACCATTTGGT
TATTCTGTTCTGTTGAAAGAGTTGATGAATTTTGCTGAAGGTAGGATTGTATTGATTTTAGAAGGGGGATATAATCTTGATTCCATTGCAAGATCAATGC
ATGCTTGCTTGGAAGTTTTGCTAAAAGACAAGCCTGTTATCAGATCCTTAGAGGCCTATCCATTTCAGTCTACATGGAATGTAATTCAAGCGGTTCGCCA
GACGTTAAGTCCCTTTTGGCCTACACTTGCATCTGAACTACCACAAGAGTTAGTCTCACAAATAGCACCCCCTCCGCATACTCTAATTTCGAGCTCTGAC
TCCGAAGATGAGGATGATAAGGGTGCAGCAAGTTTAGAAAATGTTGGAGAACTTCTTGAGGATGTCATAAAACCACTTTCCAAACTGAAAGTTGATGCTG
ATGAAGAGATTCATGTTTCTAGTACTTGGCGATCTGAATTGTCAAATGTTTATATATGGTATGCCTCGTATGGATCAAATATGTGGAAAGCAAGATTTAA
TTGCTACCTAGAAGGTGGACAGGTGGATGGTATGGTGAAGCAGTGTTCTGGTTCAGTGAACAGAACTCTGCCAAAGGAGATCATGTGGAAGACTTTCCCT
TGTGATATATTCTTTGGTCGTGATTCATCGTATTCGTGGGGTCTGGGAGGTGTTGCATTTCTTAATCCTGAAAAAATAATTGAAGGCAAGACCCACATGT
GCATGTACAAAATTTCGCTAGAGCAGTTCAATGATATTTTATTTCAGGAAAACATTTTAAGCCTTGATGCGGGCTCTCCTTTATTTGATATAACCACCTT
GAATGCTGTCTCTGACAAGGAGTTCAACTTCCTGGAGGTTGTCAAGGGTGCTTGGTATGGTAATGTTGTGTACTTAGGAAAGGAGCAGGATATTCCTGTA
ATTACCATGACGTCTTCGCTTCTTGATATTGAACGTTTCAAATCTGGGAAGCTACCATTACGTGCTCCTAATAAAGCATATGCCAACTCCTTAATTAAAG
GGTTGGTAGAGGGAGAACAACTTTCGGAGGCGGAAGCCATTGCTTACATAGAAGGTGCTGCTAAATCTTTGTGA

***Location:*** **Chromosome 3**

**4. AAD40129**

>Phvul.001G034500.2 CDS new seq
ATGCAATGGGTAAGAAAAGTGTATTTCTGGATAATTCGAGTTATCTCACATTCCATTTTCGGTGCATGCATGAAATTGGTCAACCGAGTGTCCGAGTTTA
GCAAGAACATCTCCGATTCTTCACCACCGCCATTGTTATCGCTGTCGATGTCGTCTTCTTCCGTGAACGACACCGAGGCTCTCCGGCGCAATCGCATCCT
CTCCAGCAAGCTCTACTTCGACGTGTCTCCGTCCAAGGTTCCGCTAATCTACTCCGAGTCCTACGACATAGCGTTTCTCGGCATAGAGAAACTGCATCCG
TTTGATTCGTCGAAGTGGGGACGCATTTGCCGATTCCTCGTTTCGTTTGGTATTCTCGACAAAAAATGCATCGTTGAGCCTCTGGAAGCTTCCAAGGATG
ATCTTCTAGTGGTTCACACTGAATTGTATCTGAATAGGCTGAAGGAGAGTTCAAAAGTTGCTATGATAGTTGAGGTCCCTCCCGTGGCATTAATTCCCAA
TTGTCTTGTGCAACAAAAACTTCTTTCCCCGTTCAGGAAGCAGGTTGGAGGAACTGTACTGGCTGCAAAACTTGCAAAGGAGAGAGGATGGGCCATTAAT
GTTGGAGGAGGTTTTCATCACTGCTCTGCAGAAAAAGGAGGTGGATTCTGTGCTTATGCAGATATTTCTCTTTGCATCCACTTTGCTTTTGTTCGGTTGA
ATATATCAAGGGTGATGATCATTGATCTTGATGCACATCAAGGAAACGGTCATGAAATGGACTTTGCCTATGATAGCCGAGTCTATATCTTGGATATGTA
CAATCCTGGAATATATCCTTTGGATTACGAGGCTAGAAACTACATAAATCAGAAAGTTGAAGTAAAGAGTGGGACGGTTACAGAAGAGTACCTGCAAAAA
TTAGATGAAGCACTAGAGGTTGCTGGGCGTAGGTTTAACCCTGAGTTGGTAATTTATAATGCTGGAACCGACATCCTAGAAGGAGATCCATTAGGAAGGT
TGGAGATCAGTCCTGAAGGAATTGCCCTTAGAGATGAAAAAGTTTTCCGGTTTGCTCGTGAGAGAAACATTCCCATCGTCATGCTCACTTCAGGTGGTTA
CATGAAATCTAGTGCCAGAGTGATTGCAGATTCAATAGTCAATCTTTCCAAGAAATGCTTGATAGAAACCAGTGGAGCTCCAAAGTCTTCGTGA

***Location:*** **Chromosome 1**

**5. HDT1 (AtHD2A)**

>gi|2392769|gb|AAB70032.1| putative histone deacetylase [Arabidopsis thaliana]

>Phvul.001G186300.1 CDS
ATGGAGTTTTGGGGTGTTGAAGTGAAAAGTGGACAATCTCTCAAGGTCGATCCAGGAGATGACAAGATTATCCACCTTTCAAATGCATGCCTGGGGGATG
TGACTAAGGCTAAAGGAGGTGAACTGGTAGCCCTGAACGTGAAGTTTGGTAACCAGAAGCTTGTGCTTGGAACTCTTTCTTCTGATAAATTTCCTCAAAT
ATCTTATGATTTAATATTTGAGAAGGAATTTGAGCTATCCCATAGCTGGAAAAATGGGAGTGTCTTCTTTACCGGATTCAAAGCTCAATCTCAGTCGGAG
TCTGATAATGATGAAGATTCTGACGATTTTGATGAAGATATTCCAGTTAGTGCTGCCAATGGGAAACATGAGTTTAAGGTGAAGAATGGTGTCAAACTTG
ATGAGGCTAAACAGAAGAAGATAGCAGATCCAAGGAAGAATGAAAAGGCTAAGGAAAAGGATACAAATGTCGAAGATGAAGATTCATCCGAATCTGATGA
TGATGATGATTCAAGTGAAGATAAGCCCACAGCAAATGGTGACATAGAAAGTAGTGAAGGTGATGAGGATAGTGATGATGATGATGAGGAGGAGGAGGAT
GAGGATGGTGATGACGATGAATCTGATGAGGAGACACCCAAGAAGATTGAAGAAGGCAATAAGCGGAAGATTGAATCTTCTAAGAAAACACCTGTCCCTG
AGAAAAAGGCAAAATTTGTTACTCCTCAGAAGACTGGTTAG

***Location:*** sg0.contig03923: 2769 - 5777

**6. GenBank: BAB09243.1**

>gi|9758610|dbj|BAB09243.1| transcription regulator Sir2-like protein [Arabidopsis thaliana]

>Phvul.006G057700.1 CDS
ATGTCTCTTGGGTATGCCGAGAAGCTTTCCTACATAGAAGATGTGGGCAACGTTGGAATGGTCGAATACTTCGACCCGTCTCACGTTTTGCGAGAGAAAA
TAGATCAACTTGCTATAATGATAAAAAAGAGTAAGCATCTAGTGGTATTTACAGGTGCAGGAATATCAACCTCATGTGGTATACCGGATTTTCGAGGTCC
CAAGGGAATATGGACGCTTCAGCGTGAAGGTAAAGCCTTGCCAGAAGCATCACTGCCATTTCATCGTGCAGCACCAAGTTTGACACACATGGCTTTGGTT
GAACTAGAAAAGGCTGGCATTTTGAAGTTCGTCATCAGTCAGAATGTTGATGGTCTCCATCTTCGATCCGGAATACCAAGGGAGAAATTAGCTGAGTTGC
ATGGGAACTCCTTCATGGAGACATGCCCTTCTTGTGGAGAAGAGTACTTTCGTGATTTTGAGGTAGAGACTATTGGTTTAAAGGAGACATCAAGGCGTTG
TACAGTTGCCAAATGTGGGACAAGACTTAAGGATACTGTCCTTGACTGGGAGGATGCATTGCCTCCAAAGGAGATGAATCCTGCTGAGAAGCACTGTAAA
CAGGCAGATATAGTGTTATGCTTGGGTACAAGTTTACAAATAACTCCAGCGTGCAACTTGCCTCTGAAAGCACTTCGTGGTGGGGGTAAAGTTGTTATTG
TAAATCTCCAGAAAACTCCAAAGGACAAAAAAGCCAGTTTAGTCATACATGGGTTTGCAGATAAGGTAATTGCGGGTGTCATGGACCATCTTAATATGCA
GATTCCACCATTCGTCAGGATTGATCTTTTCCAGATTGTTCTAGTGCAAGCTTTGAGCAATGATAAAAGATATGTGAACTGGACTCTCCAGATTGCCAGT
GCTCATGGACAAAGAGCGGCATTGCCTTTCATCGAGTCTGTTGAGGTCTCCTTCTTGGACAGGGAAGATTACAAAGCTGCCATTCTAGATAAGCAACCAT
TTCGGCTTAAAAGGAGAACAGCATATAACAAAGCATTTGAAATGGTTCTGAAACTCAACTTCAGTGATGGCTGTGGTTGCCCATCCCTTGAAATTGATGT
CCCGGTTGATTTTAAGGTTTCAACTGACTGTTTTGACTTTGATAAGGACTACATATTCCAAAAGCTGAGAGACAAAGCAGTTCTTGAATCAAGATGTGGT
CAGAATGGTGTTATTGAGAGAAAGACCATCTTGACACCTAGAAGTGATGTCACTACCTATGCCATTGTGACCAATGTTGTTCAGTACAGCAAAACATGTA
AAGCAGCCCTTGATTCTCTAAGTAATGGTGATCACAAAAAAAGAAAAGCTAGTGTGACTGGTACAGGATCATCTAGGAAGCGTTCAAAAGGGTCCTCCAA
GTGTAAATCCTGA

***Location:*** Chromosome 6
